# The Effects of Physiological Demands on Visual Search Behaviours During 2 vs. 1 + GK Game Situations in Football: An *in-situ* Approach

**DOI:** 10.3389/fpsyg.2022.885765

**Published:** 2022-05-30

**Authors:** Filipe Casanova, Pedro T. Esteves, Maickel Bach Padilha, João Ribeiro, Andrew Mark Williams, Júlio Garganta

**Affiliations:** ^1^Football Department, Lusófona University, Lisbon, Portugal; ^2^Centro de Investigação em Desporto, Educação Física, Exercício e Saúde (CIDEFES), Lisbon, Portugal; ^3^Research Centre in Sports Sciences, Health Sciences and Human Development (CIDESD), Maia, Portugal; ^4^Instituto Politécnico da Guarda, Guarda, Portugal; ^5^Centre of Studies and Sport Games (CEJD), Porto, Portugal; ^6^Football Department, Center of Research, Education, Innovation and Intervention in Sport (CIFI2D), Faculty of Sport, University of Porto, Porto, Portugal; ^7^Lusófona University of Porto, Porto, Portugal; ^8^Department of Health and Kinesiology, College of Health, University of Utah, Salt Lake City, UT, United States

**Keywords:** tactical performance, small-sided games, gaze behaviour, eye-tracking, Yo-Yo test

## Abstract

We examined the effect of physiological workload on gaze behaviour during defensive performance in 2 vs. 1 +goalkeeper game situations in football. Twenty-two players were assigned to either a high- or low-performing group based on a validated measure of tactical performance. A total of 12 game sequences (trials) were presented under high- and low-workload conditions. At the end of each sequence, participants were asked to indicate their perceived exertion using the Rating Scale of Mental Effort and the Borg Scale. The low- and high-workload conditions were defined when the players achieved 60 and 90% of their maximal heart rate, respectively, as per their performance in the Yo-Yo Intermittent Recovery Test. Visual search behaviours were recorded using Tobii Pro eye-movement registration glasses. Players reported higher rates of perceived exertion on the high- compared to low-workload condition. Participants in the low-performing group increased their average fixation duration and decreased the number of fixations and number of fixation locations from the low- to high-workload conditions. The low- and high-performing groups displayed different visual search strategies with regards the areas of interest fixated upon. Participants in the high-performing group focused on the *SpaceFrontPlayer*, followed by *Ball*, and *AnotherOpponent*. The low-performing group spent more time focusing on the *SpaceFrontPlayer* and *SpacePlayer* than *Ball* and *AnotherOpponent*. It appears that physiological workload and tactical expertise interact in constraining visual search behaviours in football players. Coaches and practitioners should consider ways to manipulate individual and task constraints while attending to the close interplay between physiological workload, visual behaviour, and tactical performance during practise.

## Introduction

The development of perceptual-cognitive expertise leads to a superior ability to identify and use environmental information to select and execute the most appropriate actions (Marteniuk, 1976). These skills are influenced by different interrelated constraints during performance (Williams et al., 2004; Roca and Williams, 2016), such as the mental-physiological workload, technical-tactical demands of the match, and the perceptual-cognitive skills required (Williams et al., 2011). Scientists have stressed that perceptual-cognitive skills are influenced by fatigue and match demands, with these conclusions being derived exclusively from laboratory-based protocols involving filmed simulations (e.g., Casanova et al., 2013). Casanova et al. (2013) reported that players change their gaze behaviours with increasing workload, particularly towards the end of a match in football. High-level participants fixate on significantly more locations and use more fixations of shorter duration across fatigued and non-fatigued conditions when compared with the low-level players. Yet, players increase the search time needed to pick-up relevant information from the performance environment under increasing workload conditions, particularly in less-skilled individuals. Vickers and Williams (2007) examined the effects of fatigue on gaze behaviour across different workloads in Olympic biathletes. The authors reported a change in mean fixation times and a decrement in performance at higher workloads, reflecting more efficient use of processing resources.

Thus, different physiological intensities create higher levels of stress, and ultimately fatigue (Casamichana et al., 2012), which reduces performance during a match, both technical-tactical and physiological (Gandevia, 2001; Castillo et al., 2021). Moreover, stress affects the athlete's ability to maintain attentional focus, and negatively impacts on the ability to identify relevant information to make proper decisions and perform the corresponding technical actions (Boksem et al., 2005; Smith et al., 2016). When stressed, players adapt their gaze behaviours to the different intensity demands by enrolling compensatory resources to continue to effectively process the information critical for technical skill execution (Eysenck and Derakshan, 2011).

Notwithstanding, players who display better performance levels under stress show an ability to adapt their visual strategy by allocating attention more effectively in the search for relevant information to support subsequent actions (Ward and Williams, 2003; Vickers, 2009). The observed decline in performance seems to be related to the decreased ability to identify relevant information in the game environment, as well as to suppress irrelevant cues that may be picked up during visually guided action (Gilis et al., 2008; Smith et al., 2016).

A key issue that needs to be addressed is how does workload intensity influence gaze behaviour when players perform *in-situ* rather than when viewing film simulations (Casanova et al., 2013). Previously, researchers have alluded to the fact that the inclusion of information from the performance environment in dynamic and representative experimental tasks may greatly impact the perception and action cycle when compared to typically contrived, laboratory-based tasks (Casanova et al., 2013; Dicks et al., 2017). For instance, football goalkeepers exhibit distinct gaze behaviours when they face a penalty kick during an *in-situ* task, compared to when using a joystick to respond in a laboratory setting (Dicks et al., 2010). Further efforts are needed to empirically examine whether different visual search behaviours are observed *in-situ* compared with more traditional laboratory-based tasks.

In this exploratory case study, we examined the effects induced by physiological workload on gaze behaviour during a 2 vs. 1 plus goalkeeper (GK) *in-situ* task. Also, we explore any interactions with skill level by designating participants as high- and low-performing groups based on a validated measure of tactical performance. We hypothesised that perceived effort would increase as workload increased (Pageaux and Lepers, 2016). Although previously researchers have not attempted to examine gaze behaviours under different physiological workloads using *in-situ* conditions, we expected, based on laboratory studies conducted by Casanova et al. (2013) and Vickers and Williams (2007) that players will use different gaze strategies under low- compared with high-workload conditions. The high-performing players were expected to employ less fixations to fewer locations compared with the low-performing group under both workload conditions.

## Materials and Methods

### Participants

Altogether, 22 football players (defenders and/or defensive midfielders) were separated into two groups using a median split approach based on their tactical performance measured under low-workload conditions: high-performing group (*N* = 11, age: *m* = 24.75, sd = 6.43 years; performance: *m* = 61.08, sd = 4.70%), and low-performing (*N* = 11, age: *m* = 24.75, sd = 6.43 years; performance: *m* = 53.29, sd = 2.43%). The player's defensive tactical accuracy (DTA) was assessed based on defensive tactical principles of football defined from the analysis and identification of each player's efficiency in performing the task (Teoldo et al., 2011). This assessment enables the accuracy of the player's position and movement according to a set of spatial references, as well as the analysis and categorisation of the tactical actions. We only evaluated actions related to the defensive tactical principles of play. The scores were calculated considering both negative (five-point scale) and positive actions (ten-point scale) observed during their performance in the 2 vs. 1 + GK game subphase. Previously, researchers have reported reliability values over 0.79 in the analysis of actions (Padilha et al., 2017). The G^*^Power version 3.1.9.7 was used to calculate sample size and a minimum of 16 players were required (*f* ^2^ = 0.34; α = 0.05, and β = 0.95) (Verma and Verma, 2020). The group inclusion criteria adopted were: (i) participants played at professional (Liga Portugal SABSEG) or semi-professional level (Liga 3); (ii) players reported normal levels of visual function.

The procedures were presented to the participants, who then provided informed consent. The study was approved by the Ethics Committee of the host university (protocol number CEFADE.02.2019) and all procedures were conducted in accordance with the Declaration of Helsinki.

### Experimental Task

**Figure 1:**
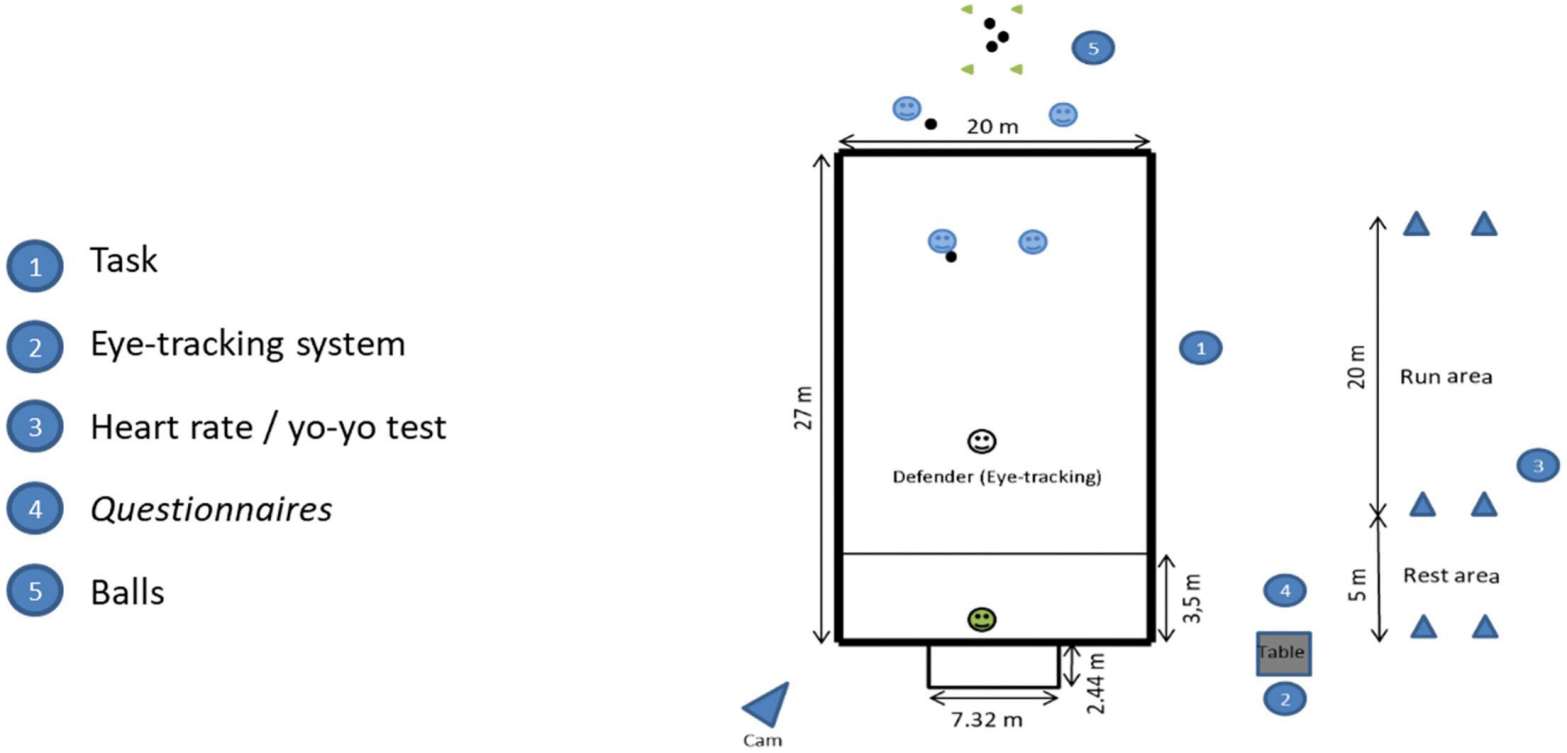
Places where each researcher positioned himself for the experimental design to be properly controlled; pitch dimensions and its procedure representativeness (2 vs. 1 + Gk).

The experimental task consisted of 12 trials involving a goalkeeper and three outfield players (i.e., 2 vs. 1 + GK). The 2 vs. 1 + GK game subphase took place on a football field with 27 m length x 20 m width (see Figure 1). These dimensions were defined according to on-field/player ratio provided by the *International Football Association Board* (Hugues, 1994). This game subphase is considered one of the most prevalent during match-play (see Ramos et al., 2019; Ribeiro et al., 2020).

The experimental task consisted of a free-play situation. The participants were evaluated only on their defensive actions (e.g., disarm, interception, tackle), whereas two attacking players (Liga Portugal SABSEG) performed the offensive actions. The trials started and finished on the command of the researcher. The trials finished either when: (i) the defender recovered ball possession; (ii) the attackers shot at goal; or (iii) when an offside or a foul was observed. To begin a new trial, the attacking players had to return to the starting point, pick up the ball, and wait for the signal to proceed. The attackers were instructed to perform an attack quickly and accurately as in a match situation.

### Workload Test Conditions

The Yo-Yo Intermittent Recovery Test (level 2) (Bangsbo et al., 2008) was used to identify the workload thresholds. There were two workload test conditions: (i) low-workload–players were required to achieve 60% of their Maximal Heart Rate (HRmax); and ii) high-workload–players were required to achieve at least 90%; both according to the age-predicted HRmax equation (Tanaka et al., 2001; Robergs and Landwehr, 2002).

### Apparatus

#### Eye-Movement Registration System

The Tobii Pro Glasses 2^®^ (Stockholm, Sweden) eye-movement system was used to record point-of-gaze onto a video image of the scene, measuring the relative position of the pupil and corneal reflection. System accuracy was recorded at 0.5° in both horizontal and vertical directions.

#### Heart Rate System

The HR was monitored continuously at 1-s intervals to provide information regarding the circulatory strain monitored by using Suunto Ambit3 peak shapphire HR system- Vantaa, Finland (Düking et al., 2016).

### Procedure

Players were asked to warm-up before the test (Karsten et al., 2016). The eye-movement system was fitted onto each participant's head. To calibrate the Tobii Pro eye-movement system, the participant had to focus on the centre of the calibration card, held in front of him, for 5 s. To ensure familiarity with the test procedure, players were given three practise trials (Williams and Davids, 1998). An Suunto Ambit3 chest band was worn by each player to measure heart frequency.

Initially, players were assigned to perform Yo-Yo Intermittent Recovery Test (level 2) to achieve at least 60% of HRmax (low-workload condition), immediately following a total of 12 game trials was prescribed to each player. After each trial, they completed the rating scale for mental effort (RSME) and the Borg scale for perceived exertion (RPE). The RSME was used to evaluate the mental effort during the task, this scale required participants to indicate the level of effort associated with task performance. The RSME ranges from 0 to 150, with three verbal anchors corresponding to 0 (*not at all effortful*), 75 (*moderately effortful*), and 150 (*very effortful*) (Zijlstra, 1993). The rating of perceived physical exertion was used with verbal anchors, which comprehended a 15-grade scale ranging from 6 (minimum effort) to 20 (maximum effort) (Borg, 1982).

Players were asked to perform the same task under the high-workload condition during which they performed Yo-Yo Intermittent Recovery Test (level 2) to achieve at least 90% of HRmax and completed the RSME and RPE scales. The individual HR values were monitored during the whole Yo-Yo test (see Figure 1). All trials were performed according to the official laws of the game.

To control for possible learning biases, four attacking players were recruited in the task. They played in alternate pairs for each trial. No feedback was provided during performance.

### Dependent Measures

#### Visual Search Behaviours

The gaze data were analysed frame-by-frame using a sampling rate of 50 Hz. A fixation was defined as a period of at least 100 ms when the eye remained stationary within 0.5° of movement tolerance (Vater et al., 2016). Visual behaviours were analysed to obtain the search rate and the percentage of viewing time (%VT) data.

##### Search Rate

Three measures were recorded to provide an indication of the search rate: mean number of visual fixations (NF); the mean fixation duration (in milliseconds; FD); and the total number of fixation locations (NFL).

##### Percentage of Viewing Time (%VT)

The %VT was defined as the proportion of time spent fixating on each of seven different display areas or areas of interest (AOIs): (i) player in ball possession (i.e., body parts-PlayerBall); (ii) space of player in ball possession (i.e., space around player and between legs-SpacePlayer); (iii) Space in front of player in ball possession (i.e., SpaceFrontPlayer); (iv) ball; (v) free space on the pitch (i.e., Space); (vi) Another opponent (i.e., opponent without ball possession); and (vii) undefined. The undefined category was excluded because the %VT in this location was <1%.

##### Reliability

Test-retest reliability comprised a 20-day interval for re-analysis to avoid any familiarity effects with the task performed using the Cohen's Kappa test (Robinson and O'Donoghue, 2007). Two independent observers were involved in this procedure. Moreover, reliability was verified through the reassessment of more than 25% of trials, as suggested in the literature (Roca et al., 2013). The intra- and inter-reliability for visual search data were above 85%. For inter and intra defensive tactical analysis, the inter-observer agreement was above 90%.

### Statistical Analysis

The distribution of data sets was analysed using Shapiro-Wilk tests. Factorial ANOVAs were performed using Group (high- and low-performing) as the between-participant factor and Workload (high- and low-workload) as the within-participant factor to examine how these factors impacted on performance. Moreover, separate factorial ANOVAs were used to analyse the RSME and RPE data respectively, with Group (high- and low-performing) as the between-participant factor and Workload (high- and low-workload) as the within-participant factor.

The different gaze data measures (NF, NFL, and FD) were analysed using separate factorial ANOVAs with Group as the between-participant factor and Workload as the within-participant factor. The %VT data across the different AOIs were analysed using a three-way ANOVA with Group as the between-participants factor and Workload and AOIs as within-participants factors, respectively. Greenhouse Geisser procedures were used to correct for violations of the sphericity assumption. Any significant main and interaction effects were followed up using Bonferroni-corrected pairwise comparisons and Bonferroni *post hoc* tests, respectively. Partial eta squared ($\eta_{\text{p}}^{2}$) values were provided as a measure of effect size for all main effects and interactions, considering that $\eta_{\text{p}}^{2}$ = 0.02 represents a small effect, $\eta_{\text{p}}^{2}$ = 0.13 represents a medium effect, and $\eta_{\text{p}}^{2}$ = 0.26 represents a large effect (Cohen, 1988). The alpha level for significance was set at *p* < 0.05.

## Results

### Rate of Perceived Exertion

There were no main effects for Group on both the RSME (*F*_1,20_ = 1.949; *p* = 0.170; $\eta_{\text{p}}^{2}$ = 0.046) and Borg scales (*F*_1,20_ = 0.163; *p* = 0.689; $\eta_{\text{p}}^{2}$ = 0.004). However, there were significant main effects for Workload on the RSME (*F*_1,20_ = 6.360, *p* = 0.016; $\eta_{\text{p}}^{2}$ = 0.137) and Borg scales (*F*_1,20_ = 18.312; *p* < 0.0001; $\eta_{\text{p}}^{2}$ = 0.314). There was no significant Group^*^workload interaction on the Borg scale (*F*_1,20_ = 1.884; *p* = 0.178; $\eta_{\text{p}}^{2}$ = 0.045) or on the RSME scale (*F*_1,20_ = 0.397, *p* = 0.532; $\eta_{\text{p}}^{2}$ = 0.010) (see Table 1). The high-performing group achieved higher values on both RSME (*p* = 0.031) and Borg scales (*p* < 0.0001) for the high- compared with the low-workload condition. In contrast, the low-performing group presented higher values only on the Borg scale during the high-workload condition when compared with the low-workload condition (*p* = 0.046). No other significant main effects were reported for scores on the RSME and Borg scales when comparing groups under low- (RSME: *p* = *0.5*91; Borg: *p* = *0.4*97) and high-workload conditions (RSME: *p* = *0.1*60; Borg: *p* = *0.2*16).

**Table 1:** Mean (m) and standard deviation (±sd) of rate of perceived exertion (RPE).

**RPE**	***Groups***	**Low-workload**		**High-workload**	
		***m***	**±*sd***	***m***	**±*sd***
RSME	High-performing	90.27^*^	27.655	115.73	26.039
	Low-performing	84.09	25.473	99.36	27.879
BORG	High-performing	14.64^*^	1.912	17.82	1.471
	Low-performing	15.18^*^	2.316	16.82	1.662

* *Significant differences between workload conditions (p < 0.05)*.

### Visual Search Rate

There were significant effects for Group on NF (*F*_1,20_ = 8.629; *p* = 0.003; $\eta_{\text{p}}^{2}$ = 0.016), NFL (*F*_1,20_ = 5.518; *p* = 0.019; $\eta_{\text{p}}^{2}$ = 0.010), and FD (*F*_1,20_ = 16.862; *p* < 0.0001; $\eta_{\text{p}}^{2}$ = 0.031). Also, there were main effects for the workload conditions on NF (*F*_1,20_ = 9.340; *p* = 0.002; $\eta_{\text{p}}^{2}$ = 0.018) and NFL (*F*_1,20_ = 9.340; *p* = 0.003; $\eta_{\text{p}}^{2}$ = 0.018). There was no significant effect for FD (*F*_1,20_ = 2.04; *p* = 0.15; $\eta_{\text{p}}^{2}$ = 0.004). There were significant Group^*^Workload interactions for NF (*F*_1,20_ = 2.482; *p* = *0.0*48; $\eta_{\text{p}}^{2}$ = 0.015) and FD (*F*_1,20_ = 14.86; *p* = *0.0*49; $\eta_{\text{p}}^{2}$ = 0.021). There were no main effects for NFL (F_1,20_ = 9.171; *p* = *0.0*56; $\eta_{\text{p}}^{2}$ = 0.007) (see Table 2). Participants in the low-performing group showed differences in the NF and NFL (NF: *p* < 0.0001; NFL: *p* < 0.0001) and increased their average FD (*p* = 0.029) between the low- and high-workload conditions. The high-performing players employed less fixations to fewer locations compared with the low-performing group on the low-workload condition (NF: *p* < 0.0001; NFL: *p* < 0.0001; FD: *p* < 0.0001). On the high-workload condition there were significant differences in FD (*p* = 0.012) but not in NF (*p* = 0.585) and NFL (*p* = 0.631) across groups.

**Table 2:** m and ±sd of visual search rate.

**Visual search**	***Groups***	**Low-workload**		**High-workload**	
**rate measures**		
		***m***	**±*sd***	***m***	**±*sd***
FD	High-performing	232.76^+^	66.54	228.45	65.16
	Low-performing	197.60^*^	54.64	215.65	69.18
NF	High-performing	16.00^+^	4.37	15.59	4.18
	Low-performing	18.30^*^	5.78	15.94	6.08
NFL	High-performing	2.55^+^	0.71	2.55	0.69
	Low-performing	2.94^*^	0.91	2.5	0.97

* *Significant differences between workload conditions (p < 0.05)*.

+ *Significant differences between groups for low-workload condition (p < 0.05)*.

### Percentage of Viewing Time

There were no significant main effects for Group (*F*_5,240_ = 0.014; *p* = *0.9*05; $\eta_{\text{p}}^{2}$ = 0.379) or Workload (*F*_5,240_ = 0.310; *p* = *0.5*78; $\eta_{\text{p}}^{2}$ = 0.001). In contrast, we observed significant differences on AOIs (*F*_5,240_ = 29.315; *p* < 0.0001; $\eta_{\text{p}}^{2}$ = 0.379). There were significant interactions for Group^*^Workload (*F*_5,240_ = 3.661; *p* = 0.003; $\eta_{\text{p}}^{2}$ = 0.071) and Workload^*^AOIs (*F*_5,240_ = 3.661; *p* = 0.003; $\eta_{\text{p}}^{2}$ = 071). The high-performing group spent more time fixating on the “SpaceFrontPlayer” than “Ball” (*p* = 0.034) and “Another Opponent” (*p* < 0.0001) under both workload conditions. Particularly, players spent less time fixating on “Another Opponent” compared with other AOIs (*p* > 0.05) in the low-workload condition. For the high-workload, the high-performing players fixated more time on “SpaceFrontPlayer” than “Ball” (*p* = 0.046) and “Another Opponent” (*p* = 0.046).

**Figure 2:**
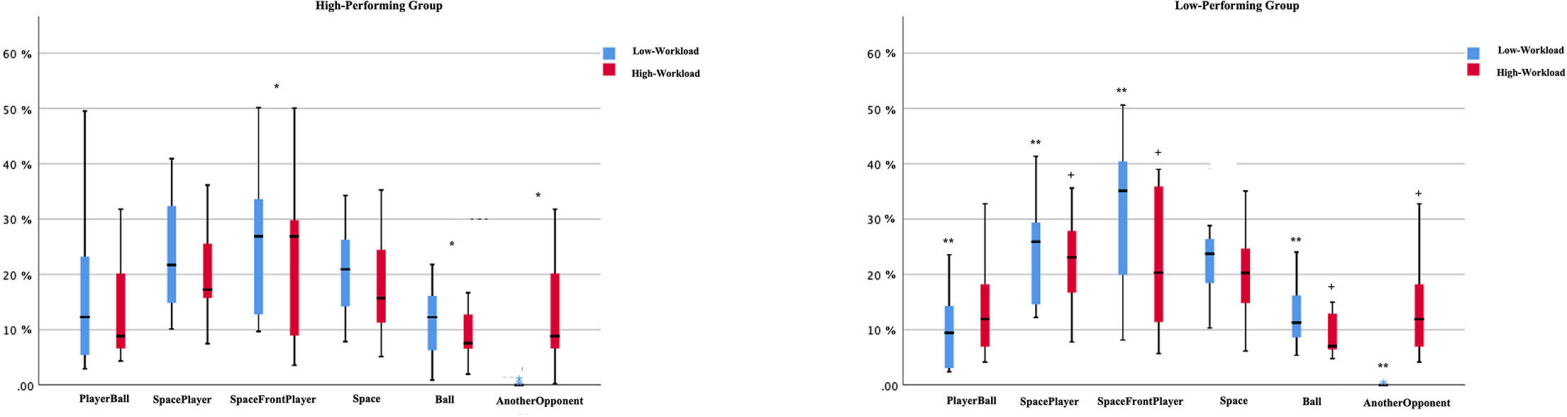
Mean percentage (%) of time spent viewing each fixation location across workload conditions for both defensive tactical accuracy groups. * Significant differences within high-performing group between SpaceFrontPlayer and Ball and AnotherOppnent in both workload conditions (*p* < 0.05). ** Significant differences within low-performing group between SpacePlayer and SpaceFrontPlayer, and PlayerBall, Ball, and AnotherOppnent in low-workload conditions (*p* < 0.05). + Significant differences within low-performing group between SpaceFrontPlayer and SpacePlayer and Ball and AnotherOppnent in high-workload conditions (*p* < 0.05).

In contrast, the low-performing group spent more time fixating on “SpacePlayer” and SpaceFrontPlayer” than “PlayerBall” (*p* = 0.001; *p* < 0.0001), “Ball” (*p* = 0.016; *p* < 0.0001) and “Another Opponent” (*p* = 0.0001; *p* < 0.0001) on low-workload conditions. In the high-workload condition they spent more time fixating on “SpacePlayer” and “SpaceFrontPlayer” than “Ball” (*p* = 0.015; *p* = 0.015) and “Another Opponent” (*p* = 0.018; *p* = 0.018) (see Figure 2).

## Discussion

We examined the effects induced by physiological workload on gaze behaviour during a 2 vs. 1+Gk *in situ* task using groups identified as high- and low-performing based on a validated measure of tactical performance. We refer to our main findings based on each measure below.

### RSME and Borg Scale

The high-performing group reported higher values on both RSME and Borg scales under high- compared with low-workload conditions. The increase in mental effort observed for the high-performing group might be linked with efforts to maintain performance by investing more resources. Players displayed higher values for RSME on the high-workload condition (high group: 90.27–115.73; low group: 84.09–99.36) (Vickers and Williams, 2007; Vater et al., 2016). Once players invest more mental effort on the task to maintain performance, the resources available to maintain optimal gaze behaviour on the task are reduced. Consequently, the potential decline in performance efficiency, may lead to a deterioration in performance effectiveness, as evident in impaired decision-making and technical-tactical performance during defensive actions (Badin et al., 2016; Smith et al., 2016).

As hypothesised, players exhibited higher values on the Borg scale under high- compared to low-workload conditions. However, there were no significant differences between groups in either condition. Previously, different physical intensities have been reported to create higher physiological stress, leading to fatigue (Casamichana et al., 2012; Casanova et al., 2013). Fatigue is associated with a decline in performance, decreasing the ability to identify relevant information in the game environment, and suppressing irrelevant cues (Gilis et al., 2008; Smith et al., 2016). Probably, for the high-performing players, a greater perceived exertion may not hold the same interconnection with the gaze behaviour processes as depicted in the mental effort evaluation. In the future, researchers could better examine the impact on mental effort and perceived exertion across different skill levels and positional roles.

### Visual Search Rate

The low-performing group displayed changes in search strategy across conditions (low-workload: FD: 197.60. NF: 18.30. NFL: 2.94; high-workload: FD: 215.55. NF: 15,94. NFL: 2.50). Notably, the low-performing group decreased the number of fixations and the mean number of fixations per location. In contrast, the high-performing group did not show significant changes on most of the gaze variables across low- and high-workload conditions (low-workload: FD: 232.76. NF: 16.00. NFL: 2.55; high-workload: FD: 228.45. NF: 15.59. NFL: 2.55), albeit they showed a tendency to decrease the number of fixations employed under high-workload conditions.

In agreement with our findings, Badin et al. (2016) suggested that when players are fatigued, their mean FD tends to increase. Such changes in visual search behaviour during the 2 vs. 1 game situations may have been influenced by task complexity and intensity, as shown by changes in effort perception. Also, such differences between groups suggest that high-performing players showed greater selectivity in searching for information cues in more relevant places than their counterparts when performing the task under low-workload conditions (Casanova et al., 2013).

Casanova et al. (2013) reported that elite players employ more fixations of shorter duration than non-elite participants and fixate on more locations at the beginning of each playing half, under low-intensity workloads. However, elite players continued to fixate on a greater number of locations, for the same amount of time, during the high-intensity physiological workload protocol. Previously, researchers have shown that different physiological workloads changes gaze behaviour (Vickers and Williams, 2007), whereas, in our study, no differences between groups were observed under high-workload conditions. While differences were evident when performing under low-workloads, the differences were less evident under high-workloads, implying that the players who show superior decision-making under low-workload may not be the ones that perform better under higher workload conditions. Moreover, during low-workloads conditions, shorter fixation durations were found in our study, which are in accordance with the previous published reports (Williams et al., 1994; Cañal-Bruland et al., 2011; Aksum et al., 2020). Participants with superior tactical expertise may have been able to react quicker and to selectively extract relevant information form the performance environment.

### Percentage of Viewing Time Per AOIs

Vaeyens et al. (2007) reported that visual search behaviours vary as a function of decision-making skill, as measured under laboratory conditions. Successful players spent more time fixating on the player in ball-possession of the ball and alternated their fixations to other AOIs (Vaeyens et al., 2007). In contrast, in our study, the high-performing players spent more time fixating on the “SpaceFrontPlayer” when compared with the “Ball”, in both conditions (low-workload: 25–12%; high-workload: 31–11%). However, the high-performing players spent more time fixating “AnotherOpponent” in the high-workload conditions. The fact that our experimental task was designed and developed *in-situ* may have contributed to these contrasting results. More research is needed to corroborate the present results, potentially involving a direct comparison between *in-situ* and laboratory-based tests.

As per Vaeyens et al. (2007), in our study players alternated their gaze between “SpaceFrontPlayer” and other locations (“PlayerBall,” “SpacePlayer,” and “Space”), although there were no significant differences among AOIs. Such changes may have been provoked by the fatigue induced by the physiological workload (Vickers and Williams, 2007; Eysenck and Derakshan, 2011) and expertise level (Casanova et al., 2013). In fact, experienced players spend more time fixating in AOIs that they considered more pertinent to their success (Williams and Davids, 1998). Moreover, it may have influenced players' search rate with clear implications on the adjustments of attentional focus away from goal-directed stimuli, potentially impairing anticipation, motor response, and decision-making (Williams et al., 2004).

Additionally, players spent more time fixating on the “AnotherOpponent” in the high-workload than the low-workload conditions. It may have been associated with physiological and mental fatigue, thus affecting processing effectiveness and changing the attentional focus from goal-directed to stimulus-driven attention (Boksem et al., 2005; Ackerman, 2011). An increase in the use of these bottom-up stimulus-driven processes under pressure might lead to threat-related attention and response tendencies (i.e., AnotherOpponent), resulting in reduced attentional control and impairment of the inhibition, shifting functions, and affecting visual perception (Eysenck and Derakshan, 2011).

Our findings provide important information for coaches when designing training sessions. Coaches need to consider how best to manipulate constraints during training to consider the key information cues used by defenders. The main challenge would be to manipulate constraints to replicate the conditions that exist during actual competitive match-play situations. Coaches could manipulate playing space, the number of players involved, and physiological workload to create realistic practise conditions. Such practise environments will allow players to engage in trial-and-error learning and lead to the development of flexible and adaptative gaze behaviours that underpin superior decision-making and creativity (Memmert and Roca, 2019).

Some limitations are worth acknowledging. First, the sample size in this study was relatively small, however, the players were mostly professional level performers. We prioritised the need to secure highly elite participants to generate more insightful findings. Second, we manipulated physiological workload by controlling the percentage of HRmax achieved by the players during both conditions. However, we did not consider the HR values during the entire trial as a continuous variable to verify the physiological impact of the task.

Our findings show that football players employ different gaze behaviours during low- and high-workload conditions. The high-performing group focused more time on the “SpaceFrontPlayer” during both conditions when compared to their counterparts, who elicited “SpaceFrontPlayer” and “SpacePlayers” areas, under the low-workload condition and, “SpaceFrontPlayer” and “SpacePlayers” under the high-workload condition. Furthermore, the results revealed that physiological workload and tactical expertise interact to drive visual search behaviours in *in-situ* game situations. From a practical perspective, coaches and practitioners should consider how best to adapt their interventions to improve visual search behaviours and performance under different workload conditions. The challenge remains how best to create practise settings that mimic the demands of competition and present desirable difficulties that enable players to search for creative solutions to task demands. Moreover, to improve the quality of instruction, particularly related to gaze behaviour, our study highlights some of the specific visual cues that may be helpful in improving defensive play. In the future, researchers need to continue to consider how gaze behaviours may change as a function of the different constraints imposed during practise and competition.

## Data Availability Statement

The raw data supporting the conclusions of this article will be made available by the authors, without undue reservation.

## Ethics Statement

The studies involving human participants were reviewed and approved by CEFADE, Faculty of Sport of University of Porto. The patients/participants provided their written informed consent to participate in this study.

## Author Contributions

FC, MBP, and AMW contributed to the conceptualisation, data collection, data analysis, and writing of the paper. PTE contributed to the data analysis and writing of the paper. JR contributed to parts of the data analysis. JG contributed to the conceptualisation and writing of the paper. All authors contributed to the article and approved the submitted version.

## Conflict of Interest

The authors declare that the research was conducted in the absence of any commercial or financial relationships that could be construed as a potential conflict of interest.

## Publisher's Note

All claims expressed in this article are solely those of the authors and do not necessarily represent those of their affiliated organizations, or those of the publisher, the editors and the reviewers. Any product that may be evaluated in this article, or claim that may be made by its manufacturer, is not guaranteed or endorsed by the publisher.
